# Gene expression analysis and the risk of relapse in favorable histology Wilms’ tumor

**DOI:** 10.1080/2090598X.2022.2127202

**Published:** 2022-09-26

**Authors:** Mariam M. Abdel-Monem, Omali Y. El-Khawaga, Amira A. Awadalla, Ashraf T. Hafez, Asmaa E. Ahmed, Mohamed Abdelhameed, Ahmed Abdelhalim

**Affiliations:** a The Center of Excellence for Genome and Cancer Research, Urology and Nephrology Center, Mansoura University, Mansoura, Egypt; b The Department of Biochemistry, Faculty of Science, Mansoura University, Mansoura, Egypt; c The Department of Urology, Mansoura Urology and Nephrology Center, Mansoura University, Mansoura, Egypt; d The Department of Pathology, Mansoura Urology and Nephrology Center, Mansoura University, Mansoura, Egypt

**Keywords:** Wilms‘ tumor, relapse, gene expression, immunohistochemistry, RNA

## Abstract

**Introduction and Objectives:**

Wilms’ tumor (WT) relapse occurs in 15% of patients. We aim to investigate the association between the expression of several genetic markers and WT relapse risk.

**Materials and methods:**

The study included 51 children treated for WT at a tertiary center between 2001 and 2019: 23 patients had disease relapse (group A) and 28 remained relapse-free after at least 2 years of follow-up (group B). Patients with syndromic, bilateral synchronous or anaplastic WT were excluded. Autologous renal tissue from 20 patients served as control. Total RNA was isolated from tumor tissue and control. Gene expression levels of WT1, HIF1α, b-FGF, c-MYC and SLC22A18 were assessed using quantitative RT-PCR and normalized to GAPDH. Immunohistochemical staining for WT1 and gene expression levels were compared between the study groups.

**Results:**

Median patient age was 3 (IQR = 2–5) years and 36 (70.6%) had stage I disease. Baseline characteristics were similar between study groups. Relapse occurred at a median of 6.8 (2.8–24.7) months, predominantly in the lungs (11/23, 47.8%). Tumors that relapsed expressed significantly higher levels of WT1, HIF1α, b-FGF and c-MYC and lower levels of SLC22A18 (p < 0.001). Strong immunohistochemical staining for WT1 was seen in 73.9% of group A and 14.29% of group B (p < 0.001). These associations retained statistical significance irrespective of patient and tumor characteristics.

**Conclusions:**

Higher expression levels of WT1, HIF1 α, b-FGF and c-MYC and lower level of SLC22A18 are associated with increased risk of WT relapse. These genetic markers can serve as future prognostic predictors and help stratify patients for treatment.

## Introduction

Tumor recurrence is known to occur in 15% of children treated for Wilms’ tumor (WT). Even with the availability of new drugs and drug combination, tumor relapse will result in a fatal outcome in 50% of patients. Several clinical risk factors have been linked to an increased risk of disease relapse including advanced stage, unfavorable histology, large tumor size, lymph node metastasis, absence of lymph node sampling, incomplete tumor resection with positive margins and local or diffuse tumor spillage [1]. Subsequent research has focused on the identification of genetic and biological factors to define tumor aggressiveness and predict relapse risk. Researchers from the National Wilms Tumor Study Group (NWTS)-5 have noted increased risk of relapse and death in patients with loss of heterozygosity (LOH) for chromosome 1p and 16q. Among patients with stage I and II favorable histology (FH) tumors, the relative risk for relapse and death was increased for LOH 1p only (RR = 2.2 for relapse; RR = 4.0 for death), for LOH 16q only (RR = 1.9 for relapse and RR = 1.4 for death), and for LOH for both regions (RR = 2.9 for relapse and RR = 4.3 for death) relative to patients without LOH at either region. The risks of relapse and death for patients with stage III and IV were higher only in patients with LOH at both loci (RR = 2.4 and 2.7 for relapse and death, respectively) [2]. High telomerase expression level [3] and gain of chromosome 1q [4] were also associated with an increased risk of WT recurrence. Despite these efforts, tumor stage, histology, patient age and LOH at 1p and 16q are the only risk factors currently used for risk stratification and clinical decision-making. These proposed factors can predict only less than one-third of all patients with tumor recurrence [5]. This indicates that other prognostic factors are involved in treatment failure and underscores the importance of identifying novel prognostic markers for more effective risk stratification and treatment planning.

In this study, we try to investigate the association between gene expression levels of WT1, HIF-1α, b-FGF, c-MYC and SLC22A18 and the risk of WT relapse. Mutations affecting the examined genes were frequently reported in other malignancies and have demonstrated a prognostic significance. We propose that these molecular markers can further refine WT treatment protocols to intensify treatment for patients at-risk of tumor relapse and subsequently improve survival. Of equal importance, less aggressive therapeutic interventions can be applied to patients with low-risk disease to minimize treatment-related morbidity and improve patient quality of life.

## Materials and methods

After IRB approval was obtained (RP.21.09.113), the database of a single tertiary center was reviewed for children ≤18 years diagnosed and treated for WT between 2001 and 2019. The exclusion criteria were bilateral synchronous WT, syndromic cases and anaplastic histology since these cases may have different genetic bases. Patient files were reviewed for demographics, imaging studies, treatment received and histopathology reports. Follow-up data were queried for the occurrence of tumor relapse, time to relapse, relapse site and survival outcome.

### Specimen collection

Formalin-fixed paraffin embedded (FFPE) blocks of radical nephrectomy specimens could be retrieved for 23 children who experienced WT relapse and represented the study group A. Histopathologic specimens of 28 age- and tumor stage-matched patients who remained free of relapse after at least 2 years of follow-up were also retrieved and comprised the study group B. Autologous normal renal tissue retrieved from 20 patients served as control. Quantitative reverse transcription PCR (qRT-PCR) was used to assess gene expression levels in tissue specimens. The expression levels of the examined genes were tested for association with WT relapse.

### Quantitative reverse transcription PCR reaction

For RNA extraction, FFPE tissue blocks were sectioned into 5 μm thick slices and placed in 2 mL tubes, and then all samples were deparaffinized. The RNeasy FFPE Kit (Qiagen, cat no. 73,504) was used for RNA extraction from FFPE tissue sections. After deparaffinization, buffer PKD was added. Samples were mixed and centrifuged for 1 min at 11,000 × g. A 10 µl of proteinase K was added to the lower clear phase. All the samples were incubated at 56°C for 15 min, then at 80°C for 15 min. The lower uncolored phase was transferred into a new 2 ml microcentrifuge tube, incubated on ice for 3 min and then centrifuged for 15 min at 20,000 × g. The supernatant was transferred to a new microcentrifuge tube. A 720 µl of 100% ethanol was added to the samples and mixed well by pipetting. A 700 µl of the sample was transferred to an RNeasy MinElute spin column and thereafter centrifuged for 15 seconds at ≥8000 × g. The flow-through was discarded. A 500 µl of buffer RPE was added to the RNeasy MinElute spin column and centrifuged for 15 seconds at ≥8000 × g. The RNeasy MinElute spin column was placed in a new 1.5 ml collection tube and 30 µl RNase-free water elutes the RNA. The concentration of RNA was assessed by nanodrop 2000c. mRNA was converted into cDNA using the High-Capacity cDNA Reverse Transcription Kit (Thermo Fisher Scientific, cat no. 4,368,814). Quantitative PCR was performed with Maxima SYBR Green qPCR Master Mix (2X) (Thermo Scientific, cat no. K0221) on the Roto-Gene real-time PCR. The mRNA expression levels of WT1, HIF-1α, b-FGF, c-MYC and SLC22A18 genes were normalized to Glyceraldehyde 3-phosphate dehydrogenase (GAPDH) as an endogenous control. The primer sequences that were used are listed in supplementary table 1. The 2− ^ΔΔCT^ method was used to calculate the relative expression of the target genes. The expression level of the studied genes was compared between the two study groups relative to the control group.

### Immunohistochemistry

The expression of WT1 was determined in the slides from WT patients using immunohistochemical staining. Briefly, 4–5 μm thick sections were deparaffinized in xylene and rehydrated in graded alcohol. For antigen retrieval, sections were placed in 0.01 M citrate buffer (pH 6.0). Endogenous peroxidase was blocked by 4% H_2_O_2_ in methanol. Sections were incubated with primary monoclonal antibody WT1 (clone: 6 F-H2, Genemed, US) in 1:100 dilution for one hour. Power-StainTM 1.0 Poly HRP conjugate (Genemed Biotakti, Inc., Cat. # 54-0022) was added for 30 min, the sections were washed with PBS, and DAB chromogen staining was applied to develop the reaction color. Slides were counterstained with H&E stain, dehydrated and mounted. Immunohistochemical analysis was performed in a blinded fashion by a single experienced uro-pathologist. The fraction of positively stained tumor cells was scored semi-quantitatively after examining under 10 high power fields (x400) for each case. Nuclear/cytoplasmic staining in >10% of tumor cells was required to define WT1 positivity. Immunohistochemistry results for WT1 were scored as weak (11–25%, 1+), moderate (26–50%, 2+) and strong (>50%, 3+) [6].

### Statistical analysis

For comparisons between groups, the chi-square test, Mann-Whitney U-test or Student t-test was used when appropriate. Kaplan–Meier curves were used to estimate survival. All tests were two-tailed with statistical significance considered at p < 0.05. Statistical analysis was performed using SPSS for Windows version 23.0 (Armonk, NY: IBM Corp).

## Results

### Patient demographics

Enrolled in the study were 51 children treated for WT between 2001 and 2019 at the study institution. Group A (WT relapse group) included 23 patients, whereas group B (relapse-free group) included 28 patients. The median age at diagnosis was 3 (IQR = 2–5) years. Patients had a median follow-up of 64 (8.3–199.6) months. The male-to-female ratio was 10:13 and 17:11 in groups A and B, respectively (p = 0.22). A total of 20 patients [9/23 (39.1%) and 11/28 (39.3%) of groups A and B, respectively (p = 0.99)] received preoperative chemotherapy. The majority of patients had stage I 36/51 (70.6%) disease. All patients were treated with radical nephrectomy and lymph node sampling through an open approach. Baseline patient characteristics were not significantly different between study groups A and B as summarized in Table 1.

**Table 1.** Baseline patient demographics.

Patient characteristics	Group A: Wilms’ tumor relapse (*N* = 23)	Group B: Wilms’ tumor relapse-free group (*N* = 28)	Control group (*N* = 20)	*p*-Value^a^
**Median age at diagnosis (range), years**	3 (0.75–8)	4 (0.5–18)	3 (1–8)	0.122
**Gender (%)**				0.22
Male	10 (43.5%)	17 (60.7%)	10 (50%)	
Female	13 (56.5%)	11 (39.3%)	10 (50%)	
**Preoperative chemotherapy (%)**	9 (39.1%)	11/28 (39.3%)		0.99
**Tumor stage (%)**				0.91
Stage I	15 (65.2%)	21 (75%)		
Stage II	5 (21.7%)	1 (3.6%)		
Stage III	1 (4.3%)	4 (14.3%)		
Stage IV	2 (8.7%)	2 (7.1%)		
**Median follow-up (range), years**	1.8 (0.25–16.6)	5.3 (2.1–10.5)		0.41

^a^Comparisons between group A (WT relapse) and group B (relapse-free group).

### WT relapse

**Figure 1.**
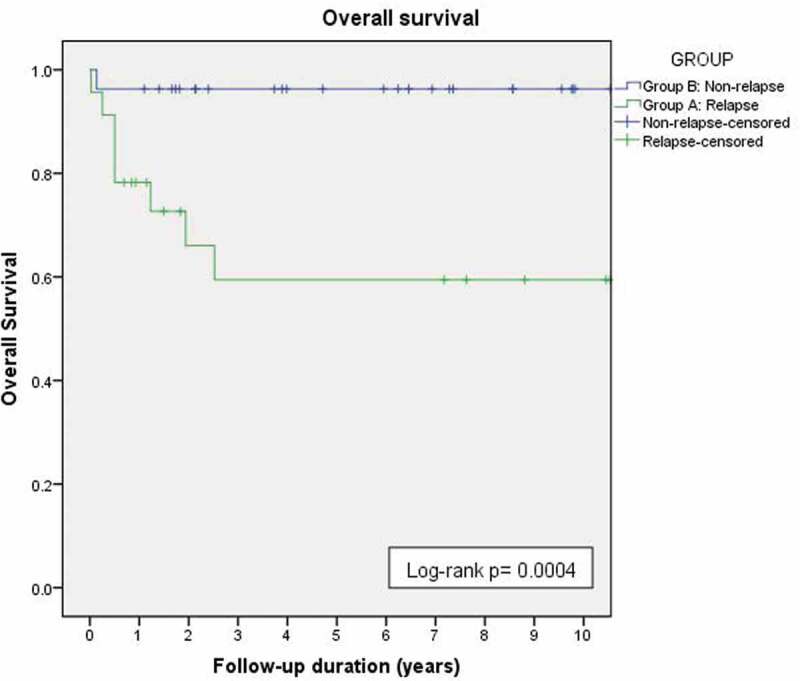
Overall survival in relapse (group A, green line) and relapse-free patients (group B, blue line) .

Among group A patients, relapse occurred at a median of 6.8 (2.8–24.7) months following surgery. A total of 11 (47.8%) patients had pulmonary relapse, 6 (26.1%) had relapse at the renal bed, 1 (4.3%) in the contralateral kidney and 5 (21.7%) relapsed at more than one site. At last follow-up, 7 (30.4%) group A patients were alive with no evidence of disease, 8 (34.8%) succumbed to their disease and 8 (34.8%) were alive with disease. The 3-year overall survival (OS) for group A and B patients was 59.5% and 96.3%, respectively (log-rank p = 0.004) (Figure 1).

### Gene expression analysis

**Figure 2.**
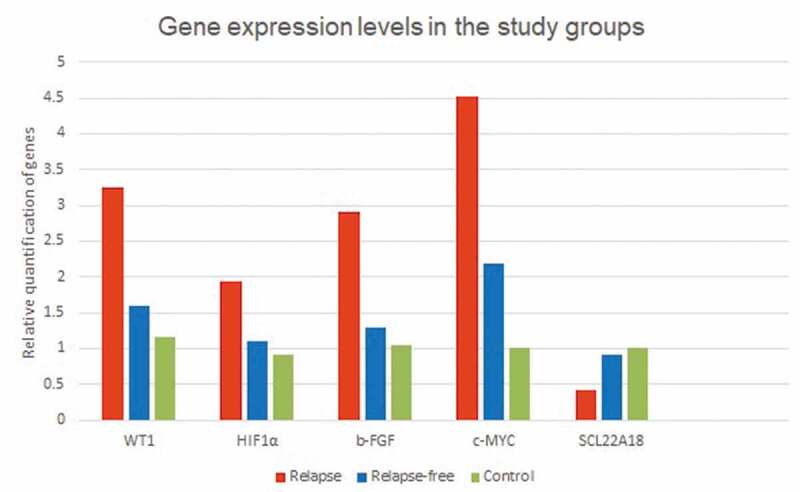
Quantitative gene expression levels in group A (Wilms’ tumor relapse), group B (relapse-free) group and controls.

Tumor tissues expressed significantly higher levels of WT1, HIF-1α, B-FGF, and c-MYC and significantly lower levels of SLC22A18 relative to autologous renal tissue. Tissue expression levels of WT1, HIF-1α, B-FGF, c-MYC were significantly higher among patients who had WT relapse (group A) relative to patients who remained free of relapse (group B). In contrast, SLC22A18 expression levels were significantly lower among group A patients (Figure 2, Table 2). These associations remained significant even after controlling for tumor stage.

**Table 2.** Gene expression levels in WT tissues among patients who had disease relapse (group A) and those who remained free of relapse (group B) relative to their levels in autologous renal tissues.

Mean gene expression levels ± SD	Control/ autologous renal tissue (*n* = 20)	Wilms’ tumor tissue (*n* = 51)	*p*-Value	Group A: Relapse group (*n* = 23)	Group B: Relapse-free group (*n* = 28)	*p*-Value
WT-1	1.16 ± 0.75	2.34 ± 1.19	<0.001	3.24 ± 1.25	1.60 ± 0.33	<0.001
HIF-1α	0.92 ± 0.53	1.47 ± 0.46	<0.001	1.93 ± 0.23	1.10 ± 0.18	<0.001
b-FGF	1.04 ± 0.57	2.02 ± 0.90	<0.001	2.90 ± 0.50	1.30 ± 0.27	<0.001
c-MYC	1.00 ± 0.12	3.23 ± 1.26	<0.001	4.52 ± 0.57	2.18 ± 0.32	<0.001
SLC 22A18	1.02 ± 0.65	0.69 ± 0.40	<0.001	0.43 ± 0.34	0.91 ± 0.31	<0.001

When comparing gene expression in patients who remained free of relapse (group B) to controls, WT patients who remained free of relapse had higher expression levels of WT-1 (*p* = 0.008), B-FGF (*p* = 0.043), and c-MYC (*p* < 0.001). Gene expression levels of HIF-1α (*p* = 0.097) and SLC22A18 (*p* = 0.128) were similar among controls and patients who remained free of relapse.

### Immunohistochemistry

**Figure 3.**
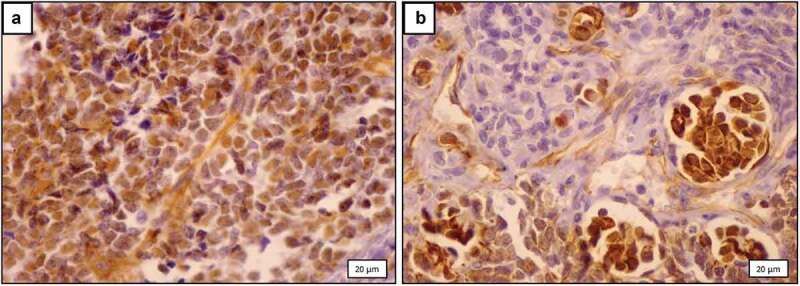
Immunohistochemical staining for WT1 showing strong positive blastemal nuclear staining in a patient who experienced tumor relapse (a) and moderate positive blastemal nuclear staining in a patient who remained free of relapse (b) (x400) .

Immunohistochemical staining for WT1 expression in relapsed patients (group A) was weak in 2(8.75%), moderate in 4(17.39%), and strong in 17 (73.9%) patients. In contrast, staining for WT1 expression in relapse-free patients (group B) was weak in 15 (53.57%), moderate in 9(32.14%), and strong in 4(14.29%) patients. The immunoreactivity to WT1 antibody was significantly higher in relapse patients compared to relapse-free patients (p < 0.001) (Figure 3).

## Discussion

Normal cellular growth requires a delicate balance between growth promoting genes, known as proto-oncogenes, and tumor suppressor genes. Tumors are characterized by uncontrolled cellular division and occur when this balance is disturbed. Perturbations causing inappropriate activation or enhanced expression of proto-oncogene, or loss of tumor suppressor gene function can lead to oncogenesis. Extensive research addressing WT tumorigenesis has resulted in the recognition of mutations affecting WT1, CTNNB1, WTX, TP53 and 11p15. Yet, about 25% of WT do not have abnormalities in any of these five genetic regions, suggesting other possible underlying genetic abnormalities that are worthy of research [7]. Identifying the genes controlling cellular growth and their products can have several potential clinical implications. First, these genes or their metabolic products can serve as tumor markers used for diagnosis, follow-up and relapse detection before it is clinically evident. Secondly, these cellular markers can indicate tumor aggressiveness and, hence, stratify patients for treatment and predict relapse risk. Importantly, these genes and the resulting metabolic pathways can be used as therapeutic targets for cancer treatment. To date, there are no reliable biological markers for WT. LOH for 1p and 16q are the only genetic markers currently used in clinical practice to stratify WT patients for treatment. In this study, we examined several biological markers in WT patients and demonstrated significant associations with tumor relapse.

WT1 gene mutations were the first genetic aberrations described in WT [8]. WT1 was originally considered a tumor suppressor gene, repressing the transcription of several growth factors and growth factor receptors including insulin-like growth factor II, insulin-like growth factor receptor, PDGF, TGF β1, PAX2, retinoic acid receptor α and EGR1. Therefore, loss of functional WT1 was thought to predispose for cancer development. More recent studies depicted that WT1 manifests both tumor suppressor and oncogenic properties. Hewitt et al. found that alternative splicing of WT1 mRNA can differentially affect its function. WT1 lacking the KTS tripeptide (lysine-threonine-serine) (WT1-KTS) was unable to repress transcription from a minimal WT1 promoter of 104 base pairs, whereas WT1 containing the KTS tripeptide (WT1+ KTS) repressed transcription from the minimal promoter [9]. Han et al. (2004) found that the overexpression of WT1 induced a significant increase in the expression of c-MYC, again suggesting a possible oncogenic role for WT [10]. In our analysis, WT1 expression levels were high in tumor tissues relative to autologous renal tissue. Further, tumors that relapsed had significantly higher expression levels and immunohistochemical staining for WT1 compared to those that remained free of relapse. In agreement with our findings, high expression levels of WT-1 were associated with poor prognosis of other malignancies such as leukemia and ovarian cancer [11].

HIF-1α is a potent stimulant for angiogenesis necessary for tumor growth and progression. Intratumor hypoxia upregulates HIF1α and other angiogenic factors, such as VEGF, that subsequently stimulate angiogenesis. Further, there is evidence that tumor cells may constitutively express HIF-1α even under normoxic conditions, suggesting that tumor cells may be capable of decoupling HIF-1α regulation from local oxygen tension. This mechanism may confer a survival advantage to tumor cells irrespective of the local oxygen levels [12]. The overexpression of HIF1α in adult and childhood tumors, including WT, has been well established. In a study by Maturu et al., all seven examined WT specimens exhibited infiltration by inflammatory immune cells with overexpression of several inflammatory markers and transcription factors, including HIF-1α, compared to normal renal tissues. HIF1α overexpression was seen in all three WT elements, predominantly in the tumor stroma. Blastemal and epithelial elements had cytoplasmic and membranous expression of HIF1α, while the stromal element had very prominent nuclear localization [13]. Karth et al. also demonstrated the co-expression of HIF-1α and VEGF in the epithelial and blastemal elements of WT specimens examined with immunohistochemistry. All examined specimens showed strong nuclear staining and 83% (15/18) demonstrated cytoplasmic expression of HIF-1α. Contrary to the results of Maturu et al., this study demonstrated little stromal expression of HIF-1α [14]. Consistent with prior reports, our study demonstrated higher expression levels of HIF-1α in tumor specimens compared to autologous normal renal tissue. Tumors that relapsed had higher expression levels than those that remained free of relapse.

Basic fibroblast growth factor (b-FGF) is another potent proangiogenic peptide that has been implicated in the progression and spread of solid tumors. It is known that b-FGF expression is directly induced by HIF1α [15]. Elevated levels of b-FGF have been observed in the urine of adult patients with renal, bladder, prostate, breast and lung cancer. Lin et al. reported elevated b-FGF levels in the urine of 70% patients with WT with significant correlation to tumor stage and relapse risk [16]. Although Skoldenberg et al. reported increased angiogenesis and increased angiogenic growth factor activity in WT specimens, they did not observe significant differences in the serum levels of b-FGF among WT patients and healthy controls. They suggested that b-FGF may be preferentially concentrated in urine to explain the high urinary levels of b-FGF in WT patients previously reported by Lin and coworkers [17]. From a clinical perspective, targeted therapy affecting FGFR signaling pathway has shown promise in treating several solid tumors including refractory cases of WT [18].

c-MYC is capable of driving cellular proliferation or apoptosis depending on other cellular signals. It stimulates cellular proliferation, glycolysis, mitochondrial biogenesis and malignant transformation. c-MYC is overexpressed in many neoplastic diseases, either due to mutation of its gene or due to the induction of its expression by a number of upstream oncogenic pathways. Both c-MYC and HIF1α act together to promote cancer cell growth and progression. c-MYC overexpression could be responsible for the increased cellular metabolism induced by malignant transformation or the result of complex metabolic changes that occur when cells turn malignant [19]. We have observed elevated bFGF and c-MYC levels in WT tissue compared to autologous renal tissue with significantly higher levels in tumors that relapsed.

SLC22A18 (solute carrier family 22, member 18) is a tumor suppressor gene located at 11p15. This genetic region is known to harbor genes coding for the Beckwith-Wiedemann syndrome and overgrowth syndromes. Abnormalities of this genetic region have been reported in a variety of cancers including 69% of WTs [7]. SLC22A18 inhibits colony formation and induces G2/M arrest. Downregulation and mutations affecting this gene have been described in glioblastoma, rhabdomyosarcoma, lung, breast and colorectal cancers. Aberrant splicing of this gene has been observed in some WT cases. Downregulation of SLC22A18 has been linked to poor prognosis of glioma and breast cancer [20]. Likewise, we found that patients who suffered WT relapse had lower expression levels of SLC22A18.

Several of our study limitations should be acknowledged including, retrospective design and possible selection bias. However, the study was conducted at a single institution and has investigated a relatively uncommon disease with relapse occurring in only a sixth of treated patients according to the contemporary treatment protocols. The small sample size has certainly limited the ability to perform multivariable analysis to adjust the tested biological markers to the well-known prognostic variables such as disease stage and LOH for 1p and 16q. This is clearly supported by the modest accuracy of other predictive models of WT relapse risk studied on larger patient cohorts with even more rigorous genetic testing [5]. Additionally, since some of the tested biological pathways could overlap, multicollinearity could compromise the quality of multivariable analysis. Some patients included in this study received preoperative chemotherapy which could have altered gene expression in the examined specimens. This study has only included patients with unilateral, non-syndromic and favorable histology WT. Therefore, we do not know if our findings could be generalized to patients with bilateral, syndromic or anaplastic tumors. Despite these limitations, the examined markers have demonstrated strong associations with the risk of disease relapse and overall survival. Future studies should be directed to unravel the underlying mutations, understand the relationship of these markers to the clinical trajectory of WT and guide potential therapeutic interventions with the hope of refining WT treatment and lowering relapse risk.

## Conclusions

Gene expression of WT1, HIF-1α, b-FGF, c-MYC and SLC22A18 is altered in WT tissues compared to normal renal tissues. Upregulation of WT1, HIF-1α, b-FGF and c-MYC and downregulation of SLC22A18 are associated with increased WT relapse risk.

## Supplementary Material

Supplemental Material
