# Supplementary material for: Gene expression analysis and the risk of relapse in favorable histology Wilms’ tumor
Source: Arab J Urol. 2022 Sep 26;21(1):45–51. doi: 10.1080/2090598X.2022.2127202 (PMC9930804; doi:10.1080/2090598X.2022.2127202)
Supplement: Supplemental Material [file TAJU_A_2127202_SM7428.docx]

Supplementary table 1: list of primer sequence for genes examined in the study

| **Gene** | **Sequence** | **Accession No.** | **Product length**  **(bp)** |
| --- | --- | --- | --- |
| WT1 | F: 5’-CAGGCTGCAATAAGAGATATTTTAAGCT-3’  R: 5’-GAAGTCACACTGGTATGGTTTCTCA-3 | [NM_024426.6](https://www.ncbi.nlm.nih.gov/entrez/viewer.fcgi?db=nucleotide&id=1889586258) | 89 |
| HIF1α | F: 5’-GTGGATTACCACAGCTGA-3’  R: 5’-GCTCAGTTAACTTGATCCA3’ | NM_001243084.2 | 115 |
| b-FGF | F: 5’AGCGGCTGTACTGCAAAAACGG3’  R: 5’CCTTTGATAGACACAACTCCTCTC3’ | [NM_001012270.2](https://www.ncbi.nlm.nih.gov/entrez/viewer.fcgi?db=nucleotide&id=1675178195) | 139 |
| MYC | F-TGTAATGGCCACATATAGCAGAAGT 3  F:5’-AAACACAAACTTGAACAGCTAC-3’  R:5’-ATTTGAGGCAGTTTACATTATGG-3’ | NM_002467.6 | 188 |
| SCL22A18 | F: 5’CCTCTGCAGCCGCAAAATC3’  R: 5’GAGAGATCCAAGCAGGAGGC 3’ | [NM_001315501.2](https://www.ncbi.nlm.nih.gov/entrez/viewer.fcgi?db=nucleotide&id=1890272267) | 187 |
| GAPDH | F: 5’TGCTGGCGCTGAGTACGTCG 3’  R: 5’TGACCTTGGCCAGGGGTGCT 3’ | [NM_001357943.2](https://www.ncbi.nlm.nih.gov/entrez/viewer.fcgi?db=nucleotide&id=1676440496) | 224 |
